# A Date of Our Own: November 13 as Bangladesh's National Physical Medicine and Rehabilitation Day

**DOI:** 10.7759/cureus.116766

**Published:** 2026-09-23

**Authors:** Mohammad Abul Kalam Azad, Md. Israt Hasan, Fatema Newaz, Md. Nadim Kamal, Taslim Uddin

**Affiliations:** 1 Department of Physical Medicine and Rehabilitation, Bangladesh Medical University, Dhaka, BGD; 2 Department of Physical Medicine and Rehabilitation, Sher-E-Bangla Medical College, Barishal, BGD; 3 Department of Physical Medicine and Rehabilitation, Kumudini Women's Medical College and Hospital, Tangail, BGD

**Keywords:** bangladesh, disability prevention, national pmr day, physical medicine and rehabilitation, rehabilitation policy

## Abstract

On November 13, 2025, the Bangladesh Society of Physical Medicine and Rehabilitation (BSPMR), the country's national professional body for the specialty, organized the first National Physical Medicine and Rehabilitation (PMR) Day in Bangladesh. The date was fixed to align with the International Physical and Rehabilitation Medicine Day. Earlier observances, held in 2017 and 2018 and revived in 2024 after a lapse of several years, followed the American Physiatry Day, whose date shifted annually.

This editorial describes BSPMR's adoption of November 13 for a national PMR observance and considers how a fixed annual date may support sustained advocacy. The case rests on a mismatch between need and provision: a rising burden of disability and population ageing, set against a specialist workforce of fewer than two physiatrists per million people, concentrated largely in Dhaka Division. We propose that future observances be used to advance five policy and service-delivery priorities: integrating rehabilitation into undergraduate medical education, extending PMR services to district-level hospitals and filling posts already sanctioned there, including rehabilitation indicators in national health information systems, securing formal state recognition of the day, and sustaining public messaging on disability prevention.

## Editorial

On November 13, 2025, Bangladesh observed its first National Physical Medicine and Rehabilitation (PM&R) Day. The event was organized by the Bangladesh Society of Physical Medicine and Rehabilitation (BSPMR), a national professional society for physiatrists that was established in 1995 as the Bangladesh Association of Physical Medicine and Rehabilitation (BAPMR) and later renamed [1]. The authors hold executive positions in BSPMR and participated in planning the observance; this editorial presents their account and perspective on its development and future role.

The day opened with a rally from the Bottola of the Bangladesh Medical University (BMU) campus in Dhaka, inaugurated by a Pro-Vice Chancellor of the university, and continued with a discussion and scientific session at BMU under the theme "Coordinated rehabilitation requires collective effort". The session was chaired by the Executive President of BSPMR, included representatives from hospitals, medical colleges, and non-governmental organizations, and received media coverage.

Speakers set out the scale of unmet need. The Bangladesh Sample Vital Statistics 2022 report, based on the Bureau's Sample Vital Registration System, a nationally representative household survey, recorded a disability prevalence of 25.5 per 1,000 population, from which approximately 4.33 million persons with disabilities are derived when applied to the census population [2]. In our view, this is likely to be a conservative estimate, since it rests on household reporting. Population aging adds to this need: nearly 9% of the population (more than 15 million people) is now aged 60 or over, a share projected to reach approximately 21% by 2050 [3]. Speakers also marked the opening of the country's first university-affiliated robotic rehabilitation center at BMU [4]. Speakers also marked the opening of the country's first robotic rehabilitation center, at BMU. Aminul Islam Bulbul, president of the Bangladesh Cricket Board, spoke as a special guest, observing that physical and mental fitness are now as decisive as technique and tactics in international cricket, and that rehabilitation specialists are central to both. 

The path to a fixed national date was longer than the single-day event suggests. Bangladesh's PMR community first observed Physiatry Day on October 13, 2017, organized by BAPMR according to society records, in parallel with the American Academy of Physical Medicine and Rehabilitation (AAPM&R), which marked the occasion on the same date [5]. The observance was repeated in 2018, jointly organized by the Department of Physical Medicine and Rehabilitation at BSMMU and BAPMR under the theme "Making life pain free and functional" [6], and again in 2022, but it was not held on a consistent annual basis. In our view, an observance without a fixed date is less likely to accumulate institutional memory. It cannot be written into an academic calendar, planned for by hospital administrations, offered to the media as a recurring fixture, or presented to a ministry as an annual point of engagement. It remains an event rather than an institution.

The resolution came from the global society. The International Society of Physical and Rehabilitation Medicine (ISPRM) was formed on November 13, 1999, and in 2023, designated November 13 as International PRM Day, commemorating both the practitioners of the specialty worldwide and the society's own founding [7]. Comparable national observances were taking shape across Asia over the same period. The Philippines fixed July 17 as National Physiatry Day through a presidential proclamation issued in 2020, first observed the following year [8]. India held its inaugural National Physical Medicine and Rehabilitation Day on July 6, 2025, led by the Indian Association of Physical Medicine and Rehabilitation (IAPMR), under the theme of preventing disability before it occurs [9].

Against this background, the interim executive committee of BSPMR resolved in 2025 to establish a National PMR Day for Bangladesh and to fix it on November 13, in alignment with the International PRM Day. The observance was established by the national professional society rather than by government proclamation; formal state recognition remains a goal, as discussed below. The choice of date was deliberate rather than convenient. Anchoring the national day to the international one situates Bangladeshi physiatry within the global rehabilitation agenda while giving the domestic community a permanent and predictable date of its own.

The case for devoting a national day to this specialty rests on a mismatch between need and provision. Rehabilitation remains among the most consistently under-resourced components of health systems, particularly in low- and middle-income countries, and Bangladesh carries a version of this burden that is intensifying rather than stabilizing [10]. Stroke and other non-communicable diseases account for a growing share of mortality and, more importantly for this specialty, of survivorship with impairment, while road traffic injury continues to produce traumatic brain and spinal cord injury in a young working population [11]. Diabetes-related complications, musculoskeletal and degenerative conditions, cerebral palsy and other childhood disabilities, and the functional consequences of an ageing population all generate demand that acute care alone does not answer [3,10,11].

Bangladesh illustrates the gap sharply. The BSPMR registry records 204 qualified physiatrists together with a further 72 members still in PMR training [12]. Society membership is not a condition of practice, so the specialist workforce is somewhat larger than the register captures; even on the most generous count it remains fewer than two physiatrists per million people, for a population above 170 million. The number has risen from 133 postgraduate-qualified physiatrists in July 2018 [10] to 204 at present [12], but not at a rate that changes that ratio in the near term. The deeper problem is not the total but its distribution. The great majority of the national PM&R workforce practices in Dhaka Division, leaving several divisions with only a handful of specialists each and some with fewer still [12].

According to records compiled by the BSPMR executive committee, more than 40 public sector institutions now provide some form of PM&R service, yet several medical colleges established in recent years hold sanctioned posts with no physiatrist in place and no service running - a gap between policy-level expansion and operational reality. The commitment on paper is not in doubt: roughly 100 junior consultant posts in PMR were sanctioned for district hospitals and *upazila* (administrative sub-district) health complexes by the Ministry of Health and Family Welfare in June 2021 (Memo No. 45.00.0000.150.15.006.21-329) [13], and, according to BSPMR records, a further tranche of specialist and faculty posts was approved in 2025. Whether they translate into services depends on recruitment, retention, and deployment, and posts created five years ago that remain unfilled are the clearest measure of the distance between the two. A patient who survives a stroke but cannot walk, dress, or return to work has not been fully treated, and across most of Bangladesh, there is no specialist within reach to complete that treatment.

A national day does not by itself close any of these gaps. What it does is create an annual, predictable occasion on which the gaps can be named publicly, to audiences that are otherwise difficult to reach. Used well, November 13 becomes the fixed point around which several longer projects can be organized. First, the integration of rehabilitation into undergraduate medical education, so that graduating physicians recognize functional outcomes as a clinical responsibility rather than a referral. Second, the extension of PMR services beyond tertiary centers in Dhaka to district-level hospitals, where most of the population lives and where rehabilitation needs largely arise, and, more immediately, the filling of the posts already sanctioned there. Third, the inclusion of rehabilitation indicators in routine national health information systems, without which the specialty remains invisible to planners. Fourth, formal state recognition of the day on the Philippine model (Presidential Proclamation No. 1017, 2020), which would give the observance administrative standing and a lead agency, though not an enforceable mandate, which would depend on its incorporation into national health or disability policy. Fifth, sustained public messaging on disability prevention - the argument, foregrounded by Indian colleagues in 2025 [9], that disability arising from several common conditions can be reduced through timely intervention, although the extent varies by condition and setting.

The second observance of National Physical Medicine and Rehabilitation (PMR) Day falls on November 13, 2026, under the theme "Developing Subspecialties, Developing PMR", and the society's first Annual Scientific Conference follows on December 5 under the theme "Strengthen Rehabilitation Services in Bangladesh, Bridging with other Subspecialties and Stakeholders". The BSPMR executive committee scheduled the two events within a month of each other and chose related themes. The day is intended to present the case for subspecialty development to the public and the press, while the conference is intended to take the same case to the profession and extend it to neighboring subspecialties and to the stakeholders who fund and staff rehabilitation services.

The establishment of a national day is a modest administrative act. A fixed annual observance may help PMR professionals communicate the role of rehabilitation in improving function, participation and quality of life, while linking national advocacy to International PRM Day. In adopting a fixed date, Bangladesh joins countries such as the Philippines and India that have established national observances for the specialty. We propose that each November 13 be assessed not by the scale of the ceremony but by measurable change since the last one, such as the number of sanctioned PMR posts filled, the number of district-level hospitals providing PMR services, the number of physiatrists per million population, and the inclusion of rehabilitation indicators in national health information systems.
